# Conserved, breed-dependent, and subline-dependent innate immune responses of Fayoumi and Leghorn chicken embryos to Newcastle disease virus infection

**DOI:** 10.1038/s41598-019-43483-1

**Published:** 2019-05-10

**Authors:** Megan A. Schilling, Sahar Memari, Meredith Cavanaugh, Robab Katani, Melissa S. Deist, Jessica Radzio-Basu, Susan J. Lamont, Joram J. Buza, Vivek Kapur

**Affiliations:** 1 The Pennsylvania State University, Huck Institutes of the Life Sciences, University Park, PA 16802 USA; 2 The Pennsylvania State University, Animal Science Department, University Park, PA 16802 USA; 3 The Nelson Mandela African Institution of Science and Technology, School of Life Science and Bioengineering, Arusha, Tanzania; 4 The Iowa State University, Department of Animal Science, Ames, IA 50011 USA; 5 The Pennsylvania State University, Applied Biological and Biosafety Research Laboratory, University Park, PA 16802 USA

**Keywords:** Innate immunity, Agricultural genetics

## Abstract

Newcastle disease virus (NDV) is a threat to the global poultry industry, but particularly for smallholder farmers in low- and middle-income countries. Previous reports suggest that some breeds of chickens are less susceptible to NDV infection, however, the mechanisms contributing to this are unknown. We here examined the comparative transcriptional responses of innate immune genes to NDV infection in inbred sublines of the Fayoumi and Leghorn breeds known to differ in their relative susceptibility to infection as well as at the microchromosome bearing the major histocompatability complex (MHC) locus. The analysis identified a set of five core genes, *Mx1*, *IRF1*, *IRF7*, *STAT1*, and *SOCS1*, that are up-regulated regardless of subline. Several genes were differentially expressed in a breed- or subline-dependent manner. The breed-dependent response involved *TLR3*, *NOS2*, *LITAF*, and *IFIH1* in the Fayoumi versus *IL8*, *CAMP*, and *CCL4* in the Leghorn. Further analysis identified subline-dependent differences in the pro-inflammatory response within the Fayoumi breed that are likely influenced by the MHC. These results have identified conserved, breed-dependent, and subline-dependent innate immune responses to NDV infection in chickens, and provide a strong framework for the future characterization of the specific roles of genes and pathways that influence the susceptibility of chickens to NDV infection.

## Introduction

Newcastle Disease Virus (NDV) is an important poultry pathogen, especially in low- and middle- income countries where over 80 percent of the poultry are reared in backyard settings with little to no veterinary care and the increased threat of exposure to pathogens^1^. Once NDV is introduced into a susceptible flock, mortality rates could be as high as 100 percent, and the disease has the potential to cause large losses in productivity for smallholder farmers who solely rely on their flocks as a source of income^1,2^. Vaccination against NDV is commonly used in high-income countries and in intensively reared poultry worldwide, but are uncommon in low- to middle-income countries, particularly in the context of backyard poultry, and hence there is a well-documented need to consider development of alternative strategies to control NDV in these settings^3^.

However, although there are low levels of vaccination in backyard poultry in these settings, many backyard poultry come in regular contact with pathogens, including NDV, in the field, but do not exhibit clinical signs of infection. This natural selection has led to locally adapted ecotypes of backyard poultry commonly reported to be naturally less susceptible to various infectious diseases, including NDV^4^. Understanding the mechanisms contributing to the relative hardiness of backyard poultry as relates to infectious disease susceptibility is important and has the potential to inform future breeding strategies to improve innate disease resistance, in chickens^4^.

Recent reports have noted differing levels of susceptibility to NDV amongst inbred Fayoumi and inbred Leghorn lines^5^. In general, the Fayoumi are considered less susceptible to multiple poultry pathogens, including *Salmonella*, *Eimeria*, Marek’s Disease Virus (MDV), and Avian Influenza Virus (AIV), as compared with Leghorn and other breeds^6–8^. While the Fayoumi originates from Egypt and is representative of the hardy backyard type poultry ecotypes common throughout the world, the Leghorn is a European origin breed that provides the foundation stock for many of the modern-day commercial egg-type chickens^9,10^. These two breeds are genetically distinct, and a recent study showed that the inbred M15.2 Fayoumi subline was less susceptible to NDV than the inbred Ghs6 inbred Leghorn subline, with reduced viral shedding in ocular secretions post-challenge^9^. However, the mechanisms contributing to these differences remain unclear.

Response to infections in birds, as in other species, involves both the innate and adaptive (humoral and cell-mediated) immune responses^11^. Given the effective and widespread use NDV vaccines, most studies of immunity to NDV have focused on the antibody and the cell-mediated responses, while much still remains to be learned about the innate immune response^12,13^. Early studies of the innate immune response of chickens to NDV have suggested high expression of genes involved in the induction of nitric oxide and the interferon response *in vitro*, *in vivo*, and *in ovo*, but much remains to be discovered, including the genetic basis and mechanisms of control of this response^14,15^.

We have recently described the chicken embryo model to examine the immune response to NDV^15^. Since chicken embryos become immunocompetent prior to hatch and remain in the protective environment of the shell, they provide a particularly tractable way to study the immune response of chickens while containing costs and reducing confounding variables often associated with animal trials^16,17^. Our preliminary analyses identified several innate immune genes that were differentially expressed between the Kuroiler and local Tanzanian ecotypes, along with evidence of breed-and subline-dependent expression in the congenic Fayoumi (M5.1 and M15.2) and Leghorn (Ghs6 and Ghs13) sublines that differ (within each line) only at the microchromosome bearing the Major Histocompatibility Complex (MHC)^5,9,15,18^. Other studies have also revealed that the Fayoumi M15.2 subline is less susceptible to NDV as compared with the Leghorn Ghs6 subline, and identified breed specific transcriptional responses to NDV infection in the trachea, lungs, and Harderian gland using an RNA-seq based approach^9,18,19^. However, these papers focused on characterizing overall pathways and mechanisms of chicks, not solely focusing on the innate immune response to NDV^9,18,19^. A closer look at the immune genes critical in the early response to NDV infection of these inbred lines is necessary to further characterize and understand their distinct responses to NDV infection.

We have here used the chick embryo model to elucidate the transcriptional response of the innate immune related genes to NDV infection in highly inbred Fayoumi and Leghorn lines. The results of our investigation provide new insights into the conserved, breed-specific, and subline-specific transcriptional innate immune responses of chickens to NDV infection. In addition, the studies provide a foundation for the future development of rational breeding strategies to improve resistance to NDV based on knowledge of innate immune responsiveness of naturally susceptible and resistant chicken ecotypes.

## Results

### The gene expression profiles of chicken embryos group into five clusters suggesting a conserved, breed-dependent, and subline-dependent response to NDV

The comparative transcriptional profiles of chicken embryos from the two inbred Leghorn sublines, Ghs6 and Ghs13, and two inbred Fayoumi sublines, M5.1 and M15.2, were examined using a custom OpenArray Technology for gene expression. The viral load of each subline and breed was assessed (Supplementary Fig. 1). The Fayoumi breed had a significantly lower viral load than the Leghorn (p-value < 0.001). When comparing the viral load between the sublines, the M15.2 Fayoumi subline had the lowest viral load and the Ghs6 Leghorn subline had the highest viral load. The table in Supplementary Fig. 1 shows the p-values for the comparisons between the different sublines.

**Figure 1:**
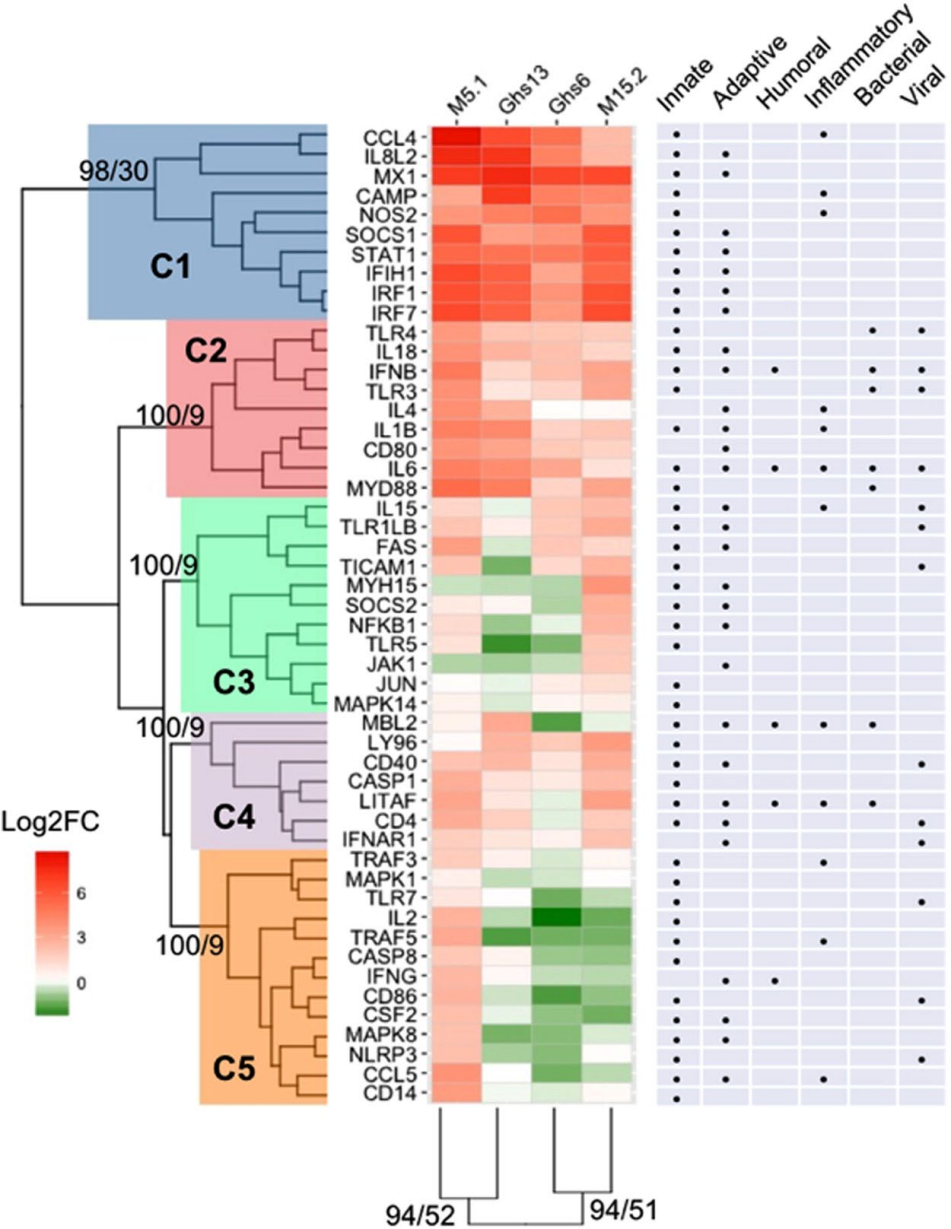
Innate Immune Gene Expression Profiles of the Fayoumi and Leghorn Sublines. The overall innate immune gene expression profiles of the two Fayoumi sublines (M5.1 and M15.2) and two Leghorn sublines (Ghs6 and Ghs13) were clustered using hierarchical clustering in R (pvclust, nboot = 1000). Each cluster is colored (C1 – blue, C2 – red, C3 – green, C4 – purple, and C5 – orange). The AU/BP values are represented at the base node of each cluster. The expression of innate immune genes, log2(Fold Change), is visualized in the heatmap (red - upregulation; green - downregulation). The functional pathways for each gene are highlighted with black dots in the table.

Hierarchical clustering grouped the innate immune related gene expression of the Fayoumi and Leghorn sublines into five clusters (Fig. 1). The approximately unbiased (AU) and bootstrap probability (BP) for all clusters are represented at the node of each major cluster. The AU value indicates there is strong clustering (AU > 95) of the genes with all AU values being statistically significant. Cluster 1 (C1), the blue cluster, contains genes that are highly upregulated in all four sublines in response to NDV, and cluster 2 (C2), the red cluster, are all upregulated but not to the extent of the blue cluster. The genes in these clusters include MX dynamin Like GTPase 1 (*Mx1*), Interferon Regulatory Factor 1 (*IRF1*) and Interferon Regulatory Factor 7 (*IRF7*), and Signal Transducer and Activator of Transcription 1 (*STAT1*) which have all been previously reported as upregulated in the response to NDV^14,15^. Clusters 3, 4, and 5, the green, purple, and orange clusters, respectively, show the genes that may be involved in breed- or subline-dependent responses. In C3 and C4, the two Leghorn sublines, Ghs6 and Ghs13, have lower expression of the genes compared to the Fayoumi sublines, with most genes in the green cluster being downregulated in the Leghorns. These genes include Suppressor of Cytokine Signaling 2 (*SOCS2*), Nuclear Factor Kappa-light-chain-enhancer of activated B cells (*NFKB*), Toll-like Receptor (*TLR5*), Janus Kinase (*JAK*), Jun Proto-Oncogene (*JUN*), and Mitogen-Activated Protein Kinase 14 (*MAPK14*). Interestingly, C5 demonstrates the genes from the M5.1 Fayoumi subline are upregulated when compared to all other sublines (M15.2, Ghs6, and Ghs13).

Hierarchical clustering of the sublines based on the individual gene expression profiles demonstrates the clustering of the Leghorn Ghs6 subline and Fayoumi M15.2 subline, while the Leghorn Ghs13 subline appears to cluster more closely with the Fayoumi M5.1 subline (Fig. 1). Although AU values are not statistically significant, there is still a trend toward considerable clustering of these sublines (AU = 94). Examining the overall gene expression profiles shows that the response of these lines may differ in a subline-dependent manner and be influenced by the MHC or other genes located on the chicken microchromosome 16, a locus that has been described to have multiple genes with a role in immunity^20^.

### The analysis reveals a core set of differentially expressed genes in the response of chicken embryos to NDV infection, as well as  breed- and subline-dependent responses

**Figure 2:**
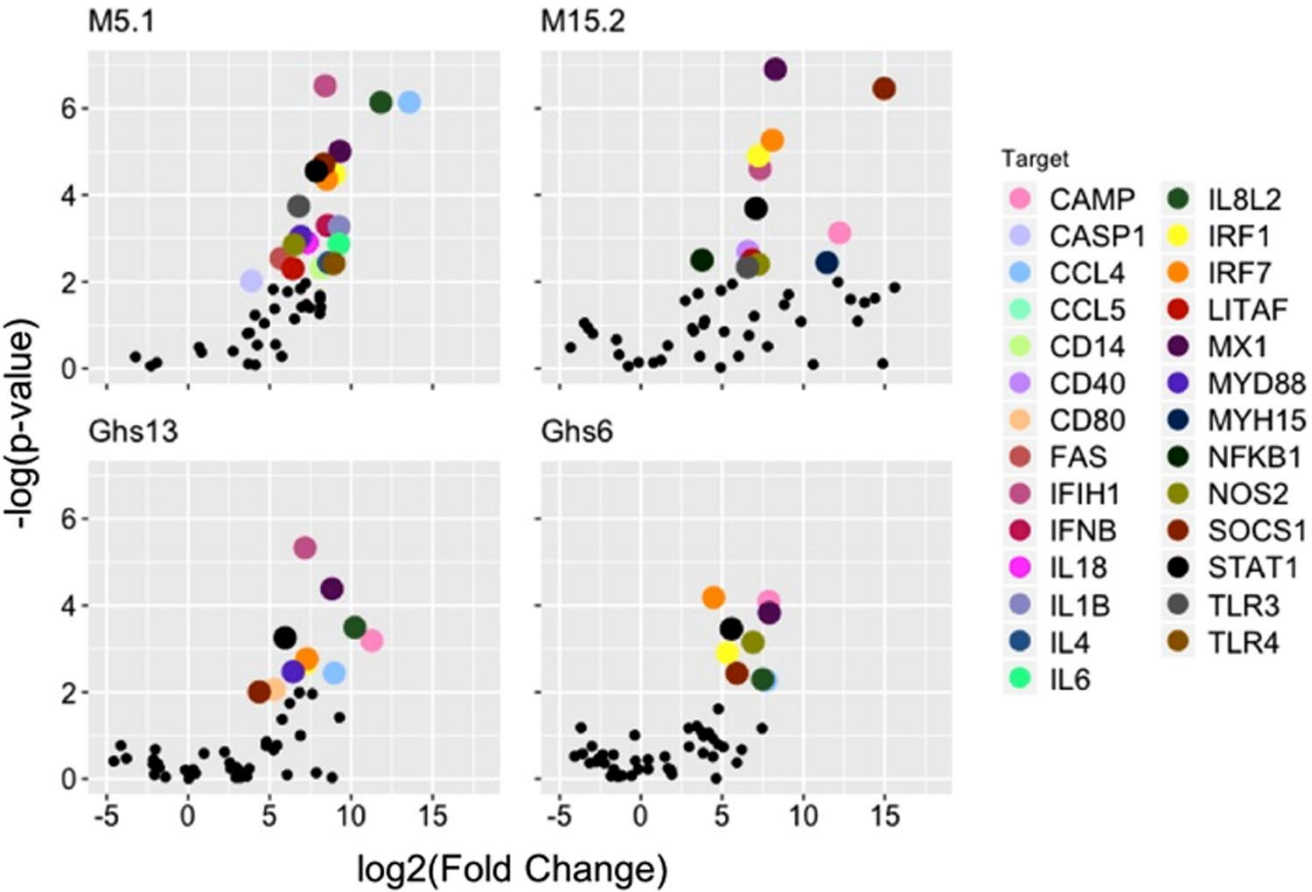
Volcano Plot of the Differentially Expressed Innate Immune Genes in each Subline. The volcano plot visualizes the log2(Fold Change) along the x-axis versus the –log(p-value) along the y-axis for the experimental versus control groups in each subline. The targets significantly expressed in the experimental group versus the controls (p-value < 0.01 or -log(p-value) > 2) are highlighted by the colors that correspond to the legend.

A volcano plot was used to visualize the gene expression profiles of each subline by comparing the normalized Ct values between the experimental versus control groups within each subline and plotting the log 2-fold change versus the negative log p-value. The significantly expressed targets, genes that are significantly expressed in the experimental group over the control group (negative log p-value > 2 equaling a p-value < 0.01), are highlighted in colors corresponding to the legend (Fig. 2). The Ghs6 subline differentially expressed nine genes, the Ghs13 had 11, the M15.2 had 13, and the M5.1 had 22 (Fig. 2). The p-values corresponding to each target within each subline is shown in Supplementary Table 1.

**Figure 3:**
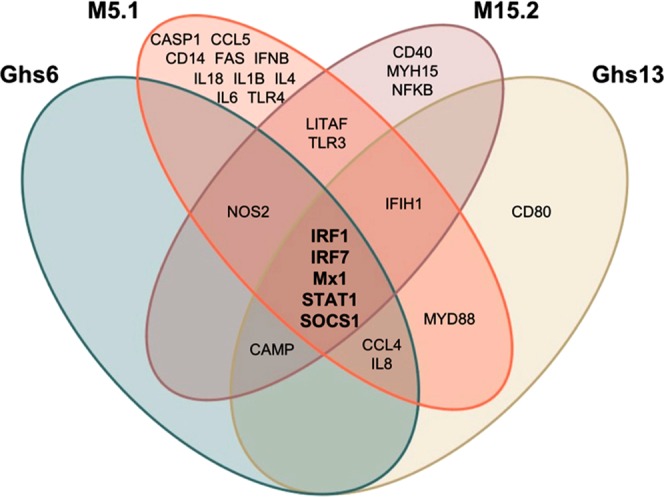
Venn Diagram Demonstrating the Common and Uncommon Differentially Expressed Innate Immune Genes in each Subline. The Venn diagram compares the targets significantly expressed in the experimental groups versus the control groups of each of the four sublines. The Ghs6 subline is represented in the green color, Ghs13 in yellow, M5.1 in orange, and M15.2 in blush.

A Venn diagram representing the significantly expressed targets (Fig. 3) and the overlaps between the four sublines identifies 5 genes (*IRF1*, *IRF7*, *Mx1*, Suppressor of Cytokine Signaling 1 (*SOCS1*), and *STAT1*) that in common across the 4 sublines (Fig. 3). Of note, the M5.1 Fayoumi subline showed the largest number of genes (10) that were unique to the subline, whereas the Gsh6 Leghorn subline had none (Fig. 3).

**Figure 4:**
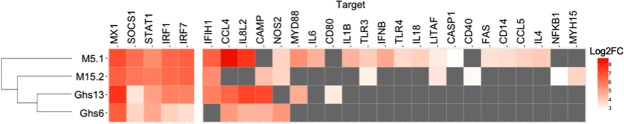
Heatmap of the Differentially Expressed Innate Immune Genes in each Subline. The fold expression changes, log2(FoldChange), range from low upregulation (light red) to high upregulation (dark red) dark red. There are no genes signifincatly downregulated in the experimental versus control groups in any of the sublines. The gray represents targets that are not differentially expressed over the controls in the respective subline. The expression profiles of the sublines were clustered using hierarchical clustering in R (pvclust, nboot = 1000).

A heatmap depicting the differential expression patterns of the differentially expressed genes for each subline is shown in Fig. 4, including the 5 core genes. The expression of the core genes ranged from 9-fold increases (Ghs13 – *SOCS1*) to 194-fold increases (Ghs13 – *Mx1*). Hierarchical clustering of expression profiles of the differentially expressed genes shows the two Leghorn sublines, Ghs6 and Ghs13 closely clustered together (AU = 99), and the M15.2 Fayoumi subline is also clustering more closely with the Leghorns (AU = 93). The M5.1, which seems to be the “high responder” and has the largest amount of differentially expressed genes, is an outlier. However, the M15.2 subline clusters closer to M5.1 with more genes that are significantly upregulated in both of these Fayoumi sublines. This contrasts the clustering using the entire dataset for the gene expression profiles (Fig. 4 versus Fig. 1), identifying both breed- (Fig. 4) and subline-dependent (Fig. 1) expression of genes in the response to NDV, and suggesting a role for the MHC in regulation of the innate immune response.

### A set of five innate immune genes drives the conserved response to NDV in different chicken lines

**Figure 5:**
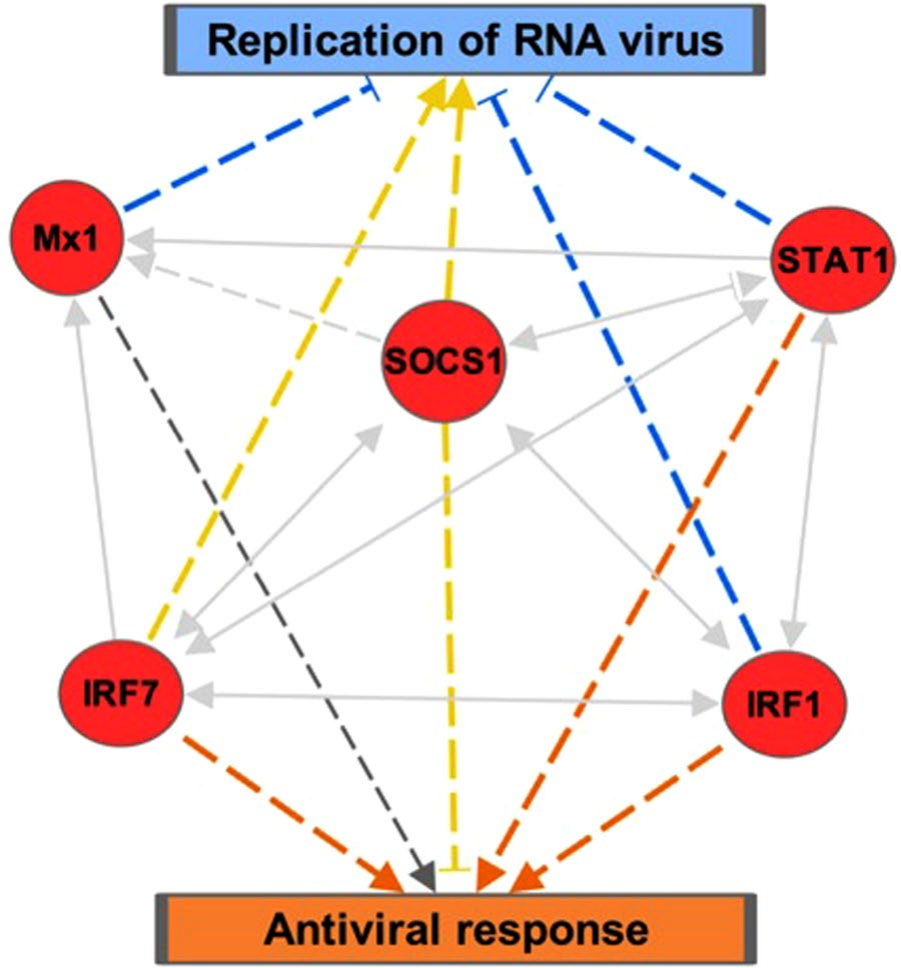
The Conserved Response of chickens to NDV infection. The gene network, including the five core genes upregulated in response to NDV, and the top Disease & Function, the antiviral response, is shown. The data were analyzed through the use of IPA (QIAGEN Inc., https://www.qiagenbioinformatics.com/products/ingenuity-pathway-analysis). The red color demonstrates the average upregulation of the genes, the light grey are the gene-gene interactions (solid = direct, dashed = indirect) the orange color shows activation of that pathway, the dark grey shows the effect is not predicted, and the yellow shows findings not consistent with downstream molecules.

The five conserved genes identified among the sublines (Fig. 3) were examined using the Ingenuity Pathway Analysis (IPA) software to define any canonical pathways or diseases/functions involved in the response to NDV. The analysis suggests that each of these five genes is involved in the interferon response (top canonical pathway, p-value 4.21 × 10^−8^) and the antiviral response (top diseases and function, p-value 1.86 × 10^−10^) (Fig. 5). The results show that *IRF1*, *IRF7*, and *STAT1* are each known to activate the antiviral response (Fig. 5, orange arrows), while *Mx1* and *SOCS1* are also both involved in the antiviral response. However, the *Mx1* gene effect is not predicted (Fig. 3, dark grey arrow) and the *SCOS1* has inconsistent findings with downstream molecules (Fig. 3, yellow arrow). The interaction network analysis shows that the expression of each of the five genes is dependent upon and interconnected with the others (Fig. 5, light grey arrows). For instance, *Mx1* directly interacts with *IRF1*, *IRF7*, *STAT1*, and itself (Fig. 5, solid light grey arrows) and the *SOCS1* gene indirectly influences the *Mx1* gene (Fig. 5, dashed light grey arrows).

### The breed-dependent response to NDV is enriched in the Fayoumi compared with the Leghorn breeds

The differentially expressed genes from the Leghorn and Fayoumi breeds are listed in Table 1 and the gene-gene network is depicted in Supplementary Fig. 2. These genes determined by examining the targets that are differentially expressed in both sublines of the respective breed. Both the Fayoumi and Leghorn responses include the five conserved genes. The responses differ by the expression of Interferon Induced with Helicase C Domain 1 (*IFIH1*), Lipopolysaccharide Induced TNF Factor (*LITAF*), Nitric Oxide Synthase 2 (*NOS2*), and Toll-like Receptor 3 (*TLR3*) in the Fayoumi and  Cathelicidin Antimicrobial Peptide (*CAMP*), C-C Motif Chemokine Ligand 4 (*CCL4*), and Interleukin-8 (*IL8*) in the Leghorn (Table 1, bold text).

**Table 1:** Breed- and Subline-Dependent Innate Immune Responses to NDV infection.

**Breed-Dependent Response**	
**Breed**	**Differentially Expressed Innate Immune Genes**
Fayoumi	**IFIH1**, IRF1, IRF7, **LITAF**, MX1, **NOS2**, STAT1, SOCS1, **TLR3**
Leghorn	**CAMP**, **CCL4**, **IL8**, IRF1, IRF7, MX1, STAT1, SOCS1
**Subline-Dependent Response**	
**Fayoumi Subline**	**Differentially Expressed Innate Immune Genes**
M5.1	CASP1, CCL4, CCL5, CD14, FAS, IFNB, IL18, IL1B, IL4, IL6, IL8, MYD88, TLR3
M15.2	CAMP, CD40, MYH15, NFKB
**Leghorn Subline**	**Differentially Expressed Innate Immune Genes**
Ghs6	NOS2
Ghs13	CD80, IFIH1, MYD88

The breed dependent responses (top 4 rows) lists the genes that are differentially expressed in both subline of the respective breed. The genes differing between the two breeds are in bold text. The subline-dependent responses (bottom 7 rows), lists the genes that are differentially expressed in each subline.

### A subline-dependent response suggests a role for the MHC in innate immunity to NDV infection

The results show considerable differences in innate immune gene expression profiles in response to NDV infection between the Fayoumi sublines. For instance, the M5.1 subline significantly upregulated 13 unique genes as compared to only 4 in the M15.2 subline. Differentially expressed genes in the M5.1 subline include multiple interleukin genes (*IL1B*, *IL4*, *IL6*, *IL8*, and *IL18*) along with other cytokines, whereas the genes upregulated in the M15.2 subline includes myosin heavy chain 15 (*MYH15*), cluster of differentiation 40 (*CD40*), *CAMP*, and *NFKB* (Table 1, Supplementary Fig. 3).

A few genes within the Leghorn sublines were also identified as subline-specific. For instance, the Ghs13 subline significantly upregulates 3 genes, *CD80*, *IFIH1*, and myeloid differentiation primary response 88 (*MYD88*). This is in contrast with the Ghs6 subline in which only *NOS2* was significantly upregulated (Table 1, Supplementary Fig. 4).

## Discussion

NDV ranks amongst the most significant diseases that limit poultry production worldwide^2^. One approach to reduce the impact of NDV infection for smallholder farmers may be through the identification and introgression through selective breeding of innate immune related genes that reduce susceptibility of chickens to NDV, with the potential of, in the long-term, improving the livability and “hardiness” of backyard poultry and, indirectly, the productivity of smallholder farmers.

To begin to understand mechanisms underlying the innate immune response of chickens to NDV, our studies have begun to identify breed- and subline-dependent differences in gene expression profiles of both inbred and outbred chicken populations with known differential susceptibilities to NDV^15^. Our results demonstrate differences in viral load amongst the lines examined here. The M15.2 Fayoumi subline had the lowest viral load, followed by the M15.2 Fayoumi subline, then the two Leghorn sublines Ghs6 and Ghs13, respectively. Overall, the Fayoumi breed also had significantly lower expression than the Leghorn. Further, the use of congenic lines suggests a role for the MHC in modulating the innate immune response to NDV. Importantly, as evident from the clustering patterns of the sublines based on the expression profiles of significantly expressed genes, the two Leghorn sublines clustered with the Fayoumi M15.2 subline. The M5.1 Fayoumi subline that was somewhat of an outlier suggests the existence of both breed- and subline-depended transcriptional responses of chickens to NDV infection (Figs 1 and 4).

The analysis identified a set of genes that were uniformly upregulated – or a “conserved response” - across all sublines. This finding is consistent with earlier reports on the immune response of chickens to NDV and other avian pathogens^7,14,15,21–25^. For instance, the response of SPF White Leghorn chickens post-virulent NDV challenge identified *Mx1*, *IRF1*, *IRF7*, and *SOCS1* were upregulated^14^. Thus, our studies with highly characterized and inbred lines confirms a role for these genes as part of the conserved response to NDV infection, as well as provides compelling evidence of conservation of the innate immune response between hatched chickens and the developing chicken embryo, further validating the use of the chick embryo model to examine the innate immune response. The five genes with the conserved responses across the sublines appear to be part of an interconnected network (Fig. 5), involving the activation of the Antiviral Response (p-value 8.16 × 10^−13^), and are likely to influence the expression of each other through multiple innate immune related pathways, including the interferon regulatory pathway, a significantly regulated canonical pathway (p-value < 6 × 10^−10^) from the Core Analysis performed in IPA. For instance, *Mx1* is known to be induced by type I and III interferons and has antiviral activity against RNA viruses^26^. *IRF1* and *IRF7* are both regulators of the interferon response and act as an activator/repressor of multiple genes and the induction of signaling cascades of pattern recognition receptors, respectively^27,28^. In contrast, *STAT1* is involved in the upregulation of genes once induced by type I, II, and III interferons and has direct interactions with the IRF genes^29^. *SOCS1* is a negative regulator of cytokines and directly regulates the JAK-STAT pathway^30^. Thus, these studies suggest that these five conserved innate immune system related genes are involved in the interferon response of chickens, and likely have significant roles in the responsiveness of chickens to NDV regardless of breed. This is consistent as well with multiple other studies that have noted the importance of interferon signaling in the response to various poultry pathogens, including Marek’s Disease (MD), Infectious Bronchitis Virus (IBV), and AIV^9,14,18,22,24,25,31,32^.

Since the conserved response seems to be important regardless of breed or infectious agent, it is tempting to speculate that the breed- and subline-dependent differences in expression patterns may provide candidate markers for further investigation that may be plausible for selective breeding for genetic improvement of response (lower susceptibility) to NDV infection.

Interestingly, when examining the genes that are differentially regulated in both Fayoumi sublines versus both Leghorn sublines, there are differences with four genes (*IFIH1*, *LITAF*, *NOS2*, and *TLR3*) in the Fayoumi and three genes (*CAMP*, *CCL4*, and *IL8*) in the Leghorn that are unique. The response of the Fayoumi and Leghorns both include the conserved genes described above. Further interaction network analysis with the breed-dependent responses revealed a significantly more interconnected pathway for the Fayoumi versus the Leghorn (Supplementary Fig. 2). This core analysis also revealed the activation of the interferon response (p-values < 6 × 10^−10^) in both breeds, consistent with the genes involved in the conserved response. *IFIH1* has been shown to be a positive antiviral gene and is also correlated with an enhanced response alongside purposive selection in some breeds^33^. The other genes upregulated in the Fayoumi all play key roles in the antiviral response as well, for example *LITAF* is a key regulator of the inflammatory response, *NOS2* has been shown to be upregulated in response to virulent NDV infection, and *TLR3* induces the interferon response^14,34,35^. The upregulation of these genes, combined with the generally higher response of the five conserved genes in the Fayoumis, may contribute to the overall enhanced response as compared to the Leghorn breed.

Although there were differences between the two breeds when examining the breed-dependent response, there are also differences seen in the subline-dependent response. Overall, the Fayoumi M5.1 subline has 12 genes unique to the subline as compared to four genes in the M15.2 subline. Interaction network analysis was also performed to examine the gene-gene networks of the sublines (Supplementary Figs 3 and 4). These responses are of interest since they may be influenced by the MHC or other genes on microchromosome 16.

Intriguingly, when investigating the gene-specific differences between the Fayoumi sublines, many interleukin genes are significantly expressed in the M5.1 subline, but not M15.2. The interleukins are secreted cytokines, particularly important in signaling the immune responses and inflammatory response^36^. One study on MD found that the expression of *IL6* and *IL18* was associated with the level of susceptibility of four Leghorn lines differing in susceptibility to MD^23^. The upregulation of the interleukins and other pro-inflammatory cytokines significantly influences many pathways as well, including the M5.1 subline represses the LXR/RXR Pathway and activates many others, including the *IL6* and *IL17* signaling pathways, the TLR pathway, and the role of pattern recognition receptors (PRRs) in recognition of virus and bacteria (Supplementary Fig. 3C). These cytokines, particularly the pro-inflammatory cytokines, play key roles in the avian immune response to pathogens and the control of infectious diseases in poultry^37^.

The differences seen between the Leghorn are one (Ghs6) and three (Ghs13) genes being subline-dependent, however, these genes may still be influencing the response to NDV (Supplementary Fig. 4). The NOS2 gene, significantly upregulated in the Ghs6 subline, is also significantly upregulated in both Fayoumi sublines, but is just below the cutoff of significance in the Ghs13 subline (p = 0.018, cutoff = 0.01). Demonstrating that this gene, although below our significance cut off, is still important in the response, and may be involved in the conserved response, which is consistent with previous studies showing the importance of the iNOS pathway in response to NDV infection^14^. The CD80 gene, significantly upregulated in the Ghs13 subline, has been found to be differentially regulated as part of the MHCII system in two meat-type broiler lines differing in susceptibility to *Salmonella* infection^21^. Although previous studies have not examined the relation of MYD88 and IFIH1 to the MHC, this study suggests a possible role for these genes in the immune response to NDV that need to be studied further.

Overall, our study is consistent with other studies regarding the core conserved gene expression response to NDV, as well as other poultry pathogens. However, the data suggest breed- and subline-dependent expression of innate immune genes that may serve as genetic markers associated with reduced susceptibility to NDV. Future studies are required to assess whether there are similar response patterns in outbred populations of chickens, as well as with more pathogenic field strains in the chick embryo. Studies associating these responses with the level of susceptibility of hatched chicks to NDV and other important avian pathogens are needed to better understand the chicken innate immune response.  This study provides a framework for future efforts to improve the health and productivity of chickens through genetic selection for reduced susceptibility to NDV and other major diseases, particularly in low- and middle income countries where NDV remains endemic and continues to represent a major threat.

## Methods

### Ethics statement and animal use

The animal use protocol was approved by the Pennsylvania State University IACUC committee (protocol number 47175). All methods were performed in accordance with the relevant guidelines and regulations outlined in this protocol. Embryonated eggs from two inbred Leghorn lines, Ghs6 and Ghs13, and two inbred Fayoumi lines, M5.1 and M15.2, were provided from the Iowa State University (Ames, IA, USA). The eggs were received and incubated at 37.5 °C, 55% humidity, rotating hourly. The eggs were temporarily removed from the incubators to candle for viability and perform inoculations with virus.

### Virus

The lentogenic LaSota strain of NDV was kindly provided by Dr. Siba Samal at the University of Maryland, College of Veterinary Medicine (College Park, MD, USA). Viral titrations were performed with the final titer of the undiluted viral suspension of 10^7^ 50% egg infectious dose (EID_50_)/mL. The viral suspension was stored at −80 °C until further use.

### Embryonated egg inoculations and tissue harvest

Embryonated eggs from each subline, at 18 days of embryonic development, were candled and the airsacs were marked. Small holes were generated just above the airsac in the eggs using an egg punch, and 0.1 mL of the viral suspension was deposited directly to the allantoic fluid. The eggs were sealed with adhesive glue and placed back in the incubators until death or removal for tissue harvest. Controls (uninfected eggs) were treated similarly and inoculated with 0.1 mL of sterile 1X phosphate buffered saline (PBS).

The eggs were removed from the incubator 72 hours post inoculation, as close to hatch as possible, and placed at 4 °C for 3–4 hours to avoid opening eggs with viable chick embryos. The chick embryos were removed from the eggs and washed with 1X PBS. The lung tissues were harvested and stored at −80 °C prior to further use.

### RNA extraction

RNA from the lung tissues of 30 experimental and 10 control embryos was extracted using the RNeasy Plus Kit (QIAGEN Inc., Germantown, MD, USA) following the recommended protocols after homogenization. The lung tissue was placed in a tube with 1 mL of RLT Lysis buffer (provided in the RNeasy kit) and 10–15 1.5 mm silica beads (Biospec Products, Bartlesville, OK, USA). The tissues were homogenized using a Mini-Beadbeater-96 (Biospec Products, Bartlesville, OK, USA) for 1 min. 600 uL of the homogenate was added to a clean microcentrifuge tube and centrifuged at 10,000 rpm for 2 min to remove any debris. The RNeasy Kit protocol was then followed with the supernatant after centrifugation.

### cDNA synthesis

cDNA synthesis was performed immediately following RNA extraction using the High Capacity cDNA Reverse Transcription Kit (Applied Biosystems, Carlsbad, CA, USA). The manufacturer protocols were followed using 2 µg of each respective RNA sample. cDNA was stored at −20 °C.

### OpenArray format and analysis

The OpenArray (Applied Biosystems, Carlsbad, CA, USA) format with 56 assays per sample and 48 samples per plate was selected (Supplementary Tables 2 and 3). The array contained 50 chicken innate immune genes, 2 viral genes, and 4 housekeeping genes. These genes were selected from our previous study in the chick embryo of different lines, other studies that found significance of these genes in the response to NDV and other pathogens, and availability of the TaqMan Gene Expression Assay from the Thermo Fisher Scientific inventory. Samples were sent to the Genomics Core Facility at the Pennsylvania State University (University Park, PA, USA) and the OpenArray was run on the QuantStudio 12 K Flex Real-Time PCR System (Applied Biosystems, Calrlsbad, CA, USA). Three technical replicates were run for each sample. The.eds files were opened using the ThermoFisher Cloud software (https://www.thermofisher.com/us/en/home/cloud.html) and exported as excel files that were used for downstream analysis.

Gene expression was analyzed using the △△Ct method comparing infected △Ct values (normalized with the Ct of the 4 housekeeping genes) with the average of the control △Ct values (normalized with the average Ct of the 4 housekeeping genes).

Figures were generated in R^38^. Hierarchical clustering was performed using pvclust package (nboot = 1000, hclust.method = “complete”, dist.method = “maximum”) and visualized with ggtree^39,40^. The volcano plot was generated using ggplot2 by plotting the log2(fold change) versus the -log(p-value)^41^, and the Venn diagram was generated with the VennDiagram package^42^.

Data were analyzed through the use of IPA (QIAGEN Inc., https://www.qiagenbioinformatics.com/products/ingenuitypathway-analysis). IPA was performed through inputting the datasets for each subline individually as well for each breed using the fold expression changes of the targets and the p-values from comparing the experimental versus control chick embryos. Core Analysis was performed for each dataset in IPA to describe the top canonical pathways and diseases/functions regulated by these genes. Comparison analyses of the core analysis for each were also generated in IPA. The gene-gene networks were generated using the Interaction Network Analysis through the build function when examining the pathways^43^. Although IPA is generally used for a larger set of genes, we were still able to examine the pathways, with a bias toward immune pathways (due to the input of only the expression of innate immune genes), that are significantly regulated post-NDV infection.

### Accession codes

The dataset is publicly available at: https://www.ncbi.nlm.nih.gov/bioproject/479417.

## Supplementary information

Supplementary Information

Supplementary Dataset 1

Supplementary Dataset 2

Supplementary Dataset 3
