# Supplementary material for: Conserved, breed-dependent, and subline-dependent innate immune responses of Fayoumi and Leghorn chicken embryos to Newcastle disease virus infection
Source: Sci Rep. 2019 May 10;9:7209. doi: 10.1038/s41598-019-43483-1 (PMC6510893; doi:10.1038/s41598-019-43483-1)
Supplement: Supplementary file 1 — Supplementary Information [file 41598_2019_43483_MOESM1_ESM.docx]

**Conserved, breed-dependent, and subline-dependent innate immune responses of Fayoumi and Leghorn chicken embryos to Newcastle disease virus infection**

**Megan Schilling^123^, Sahar Memari^12^, Meredith Cavanaugh^12^, Robab Katani^15^, Melissa S. Deist^4^, Jessica Radzio-Basu^15^, Susan J. Lamont^4^, Joram J. Buza^3^, and Vivek Kapur^1234*^**

^1^ Pennsylvania State University, Huck Institutes of the Life Sciences, University Park, PA 16802, USA

^2^ Pennsylvania State University, Animal Science Department, University Park, PA 16802, USA

^3^ The Nelson Mandela African Institution of Science and Technology, School of Life Science and Bioengineering, Arusha, Tanzania

^4^ Iowa State University, Department of Animal Science, Ames, IA 50011, USA

^5^ Pennsylvania State University, Applied Biological and Biosafety Research Laboratory, University Park, PA 16802, USA

*vkapur@psu.edu

**Supplementary Document**

**
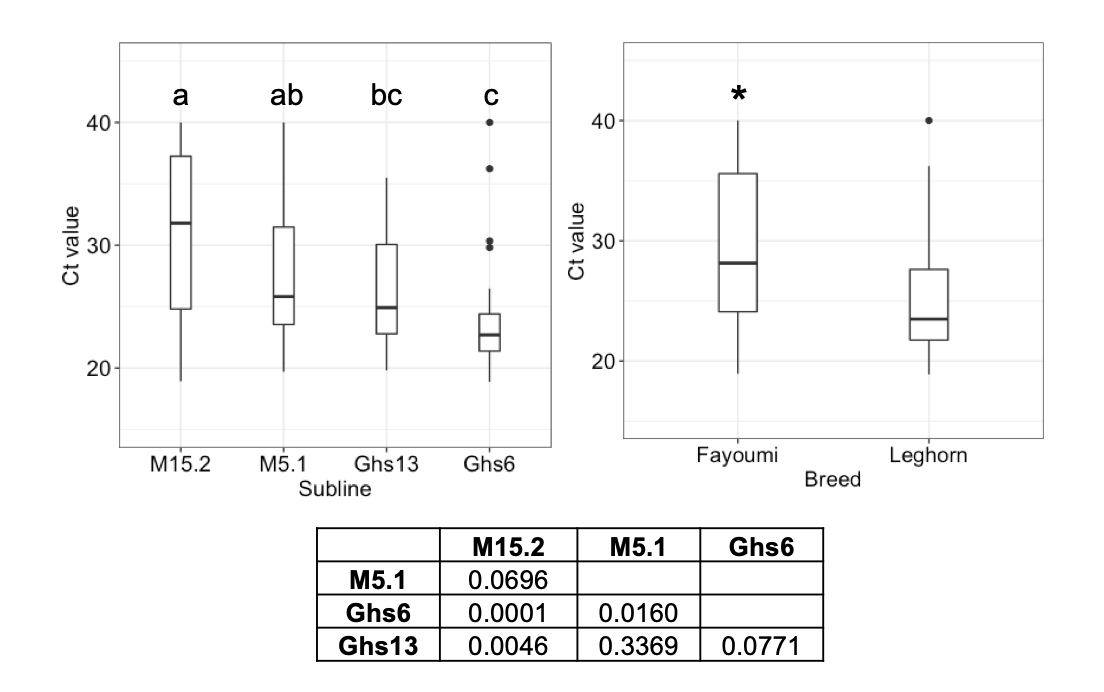
**

B

A

**Supplementary Figure 1. Newcastle disease viral load**. The viral load from the samples in each of the (A) sublines and (B) overall breed are shown in the boxplots. The boxplots show the median (middle line), Q1 and Q3 (boxes), and range (whiskers). (A) A Welch’s t-test was performed to examine the between group differences in box the sublines and the breeds. The p-values corresponding to the subline plot is in the table. (B) The viral load in the breed graph shows the Fayoumi has a statistically significant lower viral load than the Leghorn (p-value < 0.001).


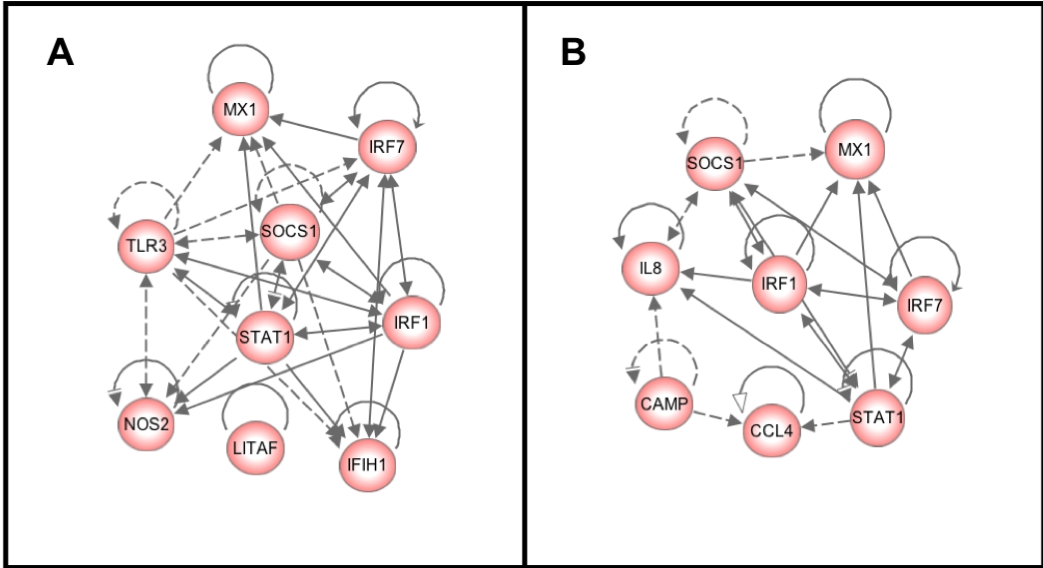


**Supplementary Figure 2. Breed-dependent innate immune gene pathways.** The gene-gene network of the differentially expressed genes that are present in (A) both Fayoumi sublines and (B) both Leghorn sublines. Direct gene-gene interactions are represented by the solid grey lines and indirect gene-gene interactions by the dashed lines. The networks were generated through the use of IPA (QIAGEN Inc., https://www.qiagenbioinformatics.com/products/ingenuity-pathway-analysis).


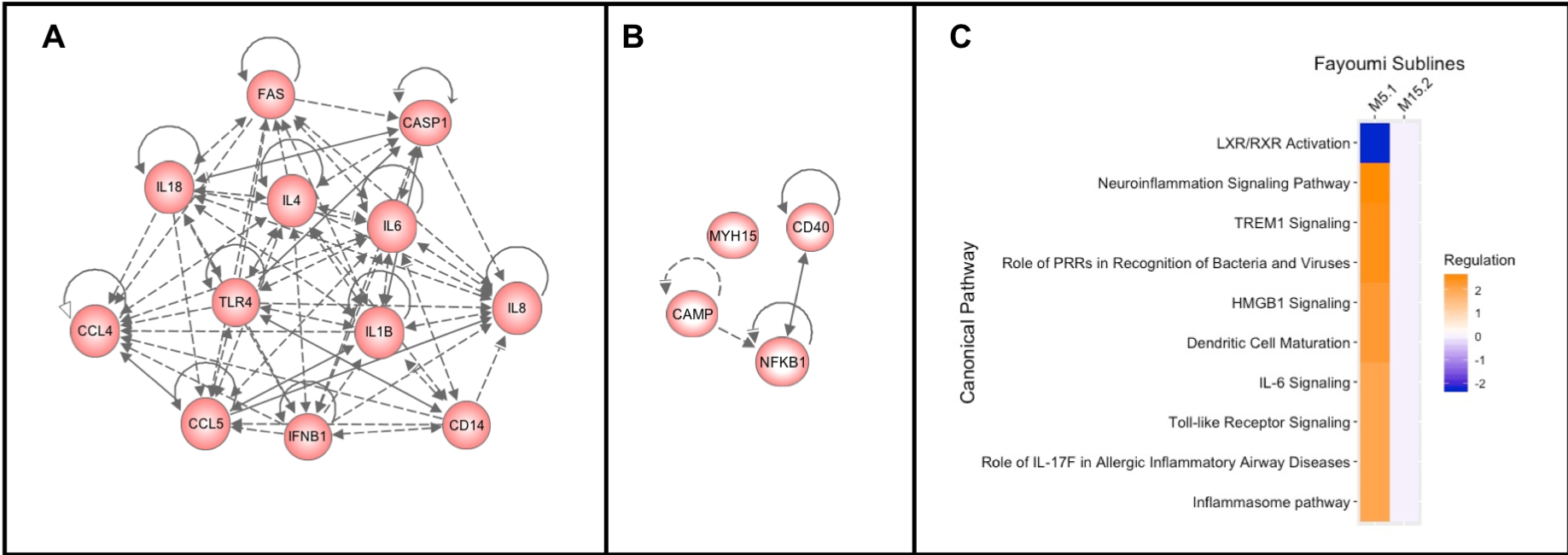


**Supplementary Figure 3. Fayoumi subline-dependent innate immune gene pathways.** The gene-gene network of the differentially expressed genes that are unique to the (A) M5.1 subline and (B) M15.2 subline. Direct gene-gene interactions are represented by the solid grey lines and indirect gene-gene interactions by the dashed lines. (C) Pathway analysis was performed to examine canonical pathways regulated by the expression of the genes unique to each subline. The M15.2 subline, with only four genes differentially expressed, did not have enough data to generate pathways influenced by the expression of the genes. The networks and analyses were generated through the use of IPA (QIAGEN Inc., https://www.qiagenbioinformatics.com/products/ingenuity-pathway-analysis).


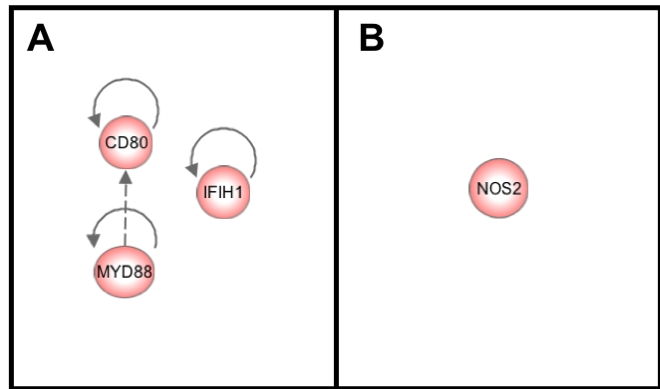


**Supplementary Figure 4. Leghorn subline-dependent innate immune gene pathways.** The gene-gene network of the differentially expressed genes that are unique to the (A) Ghs13 subline and (B) Ghs6 subline. Direct gene-gene interactions are represented by the solid grey lines and indirect gene-gene interactions by the dashed lines. The networks were generated through the use of IPA (QIAGEN Inc., https://www.qiagenbioinformatics.com/products/ingenuity-pathway-analysis).
